# Conservation and the genomics of populations, 3rd edition, Allendorf F. W., Funk W. C., Aitken S. N., Byrne M., Luikart G.

**DOI:** 10.1111/eva.13499

**Published:** 2022-10-31

**Authors:** Gernot Segelbacher

**Affiliations:** ^1^ Wildlife Ecology and Management University of Freiburg Freiburg im Breisgau Germany

It has been 15 years since the first edition of ‘Conservation and the Genetics of Populations’ was published in 2007. Since then, molecular tools have been rapidly advancing, and the number of scientific papers using large genomic datasets is steadily increasing. The authors of the book present an informative updated graph in figure 4.1. on the development of genetic markers over time, and we can expect the sequencing of genomes playing an even more important role in the coming years. Thus, the new title replacing the ‘genetics’ by ‘genomics’ seems to be timely. The authors now added a full new chapter on population genomics, giving a brief overview on current sequencing technologies and workflows. For many readers with a conservation background, this is a helpful overview without getting lost in too much detail. What else is new? The book is not only thicker (746 pages compared with 664 of the first edition) but also heavier (now 1.68 kg, compared with the 1.29 kg of the first edition), and coloured figures and photos have been added. More importantly, the book is more diverse: The author team has been expanded over time, with Chris Funk and Margaret Byrne added for the 3rd edition, Sally Aitken added for the 2nd edition authors team and the two original authors Fred Allendorf and Gordon Luikart. The authors also included additional guest authors (*n* = 42). These provided case studies with wide taxonomic coverage and in various ecosystems something that is a strength of the book. Readers can get a good impression of the underlying concepts of the field and how genomic tools can be applied in real‐world scenarios.

The outline of the book remained similar in the first part including a general introduction on methods and the role of genetic tools in conservation. The second part explains the principles of population genetics concisely and has been updated with more actual examples. The former third part has now been split into two parts, namely ‘evolutionary response to anthropogenic changes’ and ‘conservation and management’. The chapters of the previous 2nd edition have been rearranged across these two new parts. However, I felt the distinction in the two parts a bit difficult to follow, as chapters on hybridisation and invasive species are not anymore in the ‘management’ chapter but still have management‐related aspects. There is a new chapter on genetic monitoring (formerly included in the species identification and now expanded) and the book ends with a dedicated chapter on conservation genetics in practice (written by Helen Taylor). This last chapter is one of the highlights of the book for me. It links to applied management and how we can potentially overcome the conservation genetics gap. This chapter also makes a good ending by illustrating the rationale of the book and why the book is essential for the conservation community. Genetic diversity is receiving more attention in management internationally and nationally, and this book gives a number of illustrative case studies. However, while the number of scientific papers in the field of conservation genetics and management is still increasing, the integration of genetic diversity on the policy level is still low.

So who should read this book? A section in the end asks, ‘how do I become a conservation geneticist?’—and it is the future generation of experts in the field that should use this book as a starting point. Students and teachers will definitely benefit from the updated literature and case studies and the extensive glossary provided. Today, many studies apply the latest genomic tools, and often students are left in spending most of their days with coding or bioinformatics analyses. There is a risk to forget about the underlying principles of population genetics on the one side and the management implications on the other. I do think the book nicely complements the theory with real‐world examples and provides important background reading, which I would recommend to each student starting a conservation genomics project. Informed conservationists will also benefit from the overview on what is possible with today's technologies.

While I applaud the authors for undertaking such massive tasks in updating the book, I found a few things, which will likely get more attention in the future: The added complexity of data storage, data standardization and international collaborations is getting more and more important. We already know from the times of microsatellites that it is difficult to compare results among labs or countries—more data that are available will need clear agreements on protocols and standards across the world. This is especially true for the exploding applications of metabarcoding and eDNA approaches for measuring biodiversity, which gets relatively little attention in the book.

Saying all this, I was surprised that the quality of pictures and photographs was often quite low in printing. It also seems that many people had to wait very long to obtain the book from the new publisher. Electronic access is also very restricted and limited. I do think the book would have an even higher impact and be more relevant for many students and practitioners across the world if it would be more accessible.

Molecular tools are still rapidly developing and technologies allow even the use of portable sequencing tools in remote areas. The authors did an excellent job in summarizing the recent developments and included the most recent discussion in the literature (such as highlighting the field of macrogenetics). Obviously, the field will move forward rapidly, and with more and more genomes of additional species available, more opportunities for analyses exist. At the same time, the vast amount of data adds an additional level of complexity, so that collaborations and communication with managers will become even more important. The times when a single scientist was sampling, analysing, interpreting data and maybe even giving recommendations all in one person are gone, and the field of conservation genetics/genomics is now a true collaborative and team effort. The authors have shown how such a successful collaboration can look like.

